# Twelve-year-old boy with decreased vision in his right eye

**DOI:** 10.4103/0974-620X.57319

**Published:** 2009

**Authors:** Ahmed Al-Hinai

**Affiliations:** Department of Ophthalmology, Sultan Qaboos University Hospital, Muscat, Sultanate of Oman

## Questions:

**Figure 1:**
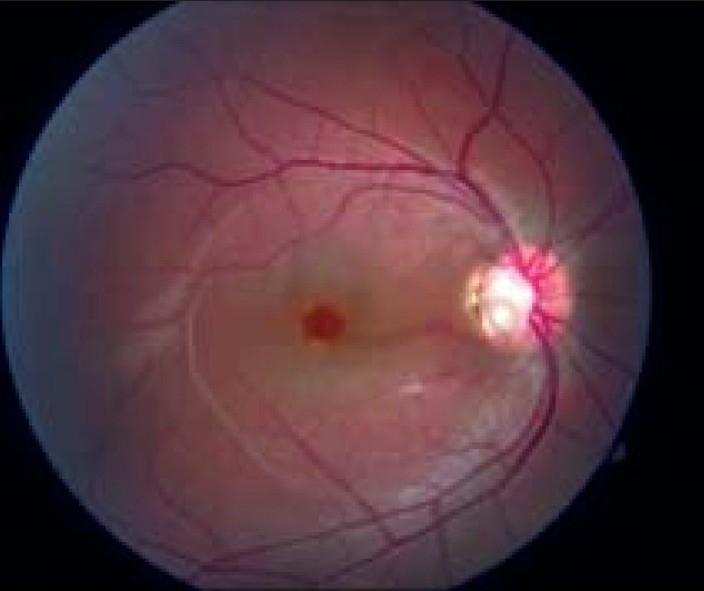
Fundus photograph OD

A 12-year-old Caucasian boy complains of decreased vision in his right eye for few months. The fundus picture is shown in Figure 1. Otherwise, he is healthy.

1. What is the diagnosis?
2. Why his vision is poor in his right eye?

## Answers

1. Right Eye Optic Pit
2. Serous macular detachment

## Optic Pit

Optic pit is a rare congenital optic disc anomaly (1 in 11 000). It was first described in 1882 by Wiethe. It is a round to oval depression in the optic disc, and its color is gray to white due to glial remnants. Commonly, it is located at inferotemporal portion of the disc [Figure 1]. Optic pits are typically unilateral and tend to occur in patients with large optic discs. They occur in a sporadic pattern but autosomal-dominant pattern of inheritance had been described. Visual acuity is usually unaffected unless serous macular detachment develops, which can occur in 25–75% of patients. Typically, there is no other ocular or systemic involvement.

Three possible theories for source of the fluid in serous macular detachment associated with optic disc include: 1) liquefied vitreous from vitreous cavity, 2) cerebrospinal fluid from subarachnoid space, 3) leaking blood vessels at optic pit base. However, Lincoff suggested in 1988 that retinal schisis initially forms in direct communication with the optic pit.

A successful treatment for the serous macular detachment secondary to optic pits is still to be discovered. On the other hand, different therapies had been tried, and these include: oral corticosteroids, laser photocoagulation and pars plana vitrectomy with or without gas tamponade. In addition, pars plana vitrectomy with usage of tissue glue (Tisseel) to seal the optic pit, and gas tamponade, has also been tried with some benefit.

